# Homoeologous duplicated regions are involved in quantitative resistance of *Brassica napus* to stem canker

**DOI:** 10.1186/1471-2164-15-498

**Published:** 2014-06-19

**Authors:** Berline Fopa Fomeju, Cyril Falentin, Gilles Lassalle, Maria J Manzanares-Dauleux, Régine Delourme

**Affiliations:** UMR1349 IGEPP, INRA, BP35327, 35653 Le Rheu, France; UMR1349 IGEPP, AGROCAMPUS OUEST, BP35327, 35653 Le Rheu, France

## Abstract

**Background:**

Several major crop species are current or ancient polyploids. To better describe the genetic factors controlling traits of agronomic interest (QTL), it is necessary to understand the structural and functional organisation of these QTL regions in relation to genome duplication. We investigated quantitative resistance to the fungal disease stem canker in *Brassica napus,* a highly duplicated amphidiploid species, to assess the proportion of resistance QTL located at duplicated positions.

**Results:**

Genome-wide association analysis on a panel of 116 oilseed rape varieties genotyped with 3228 SNP indicated that 321 markers, corresponding to 64 genomic regions, are associated with resistance to stem canker. These genomic regions are relatively equally distributed on the A (53%) and C (47%) genomes of *B. napus*. Overall, 44% of these regions (28/64) are duplicated homoeologous regions. They are located in duplications of six (E, J, R, T, U and W) of the 24 ancestral blocks that constitute the *B. napus* genome. Overall, these six ancestral blocks have 34 duplicated copies in the *B.napus* genome. Almost all of the duplicated copies (82% of the 34 regions) harboured resistance associated markers for stem canker resistance, which suggests structural and functional conservation of genetic factors involved in this trait in *B. napus.*

**Conclusions:**

Our study provides information on the involvement of duplicated loci in the control of stem canker resistance in *B. napus*. Further investigation of the similarity/divergence in sequence and gene content of these duplicated regions will provide insight into the conservation and allelic diversity of the underlying genes.

**Electronic supplementary material:**

The online version of this article (doi:10.1186/1471-2164-15-498) contains supplementary material, which is available to authorized users.

## Background

Polyploidy or whole genome duplication (WGD) is an important phenomenon that has occurred during speciation and diversification of most plant species [1, 2]. It is estimated that 70% of angiosperms are polyploid [1, 2]. Polyploidy results from either the duplication of a same genome (autopolyploidy) or the interspecific hybridisation of genomes of two related species (allopolyploidy). The duplicated regions undergo important changes that can cause structural, functional and/or regulatory modifications to the duplicated genes. Various reports have suggested mechanisms to explain the evolution of duplicated genes including the gene balance hypothesis, diploidization, and neo- / sub- functionalization [1–4]. It is expected that these evolutionary processes might play a fundamental role in the diversification of the genes underlying complex traits. Various studies, mainly in allopolyploid plant species, have highlighted the involvement of duplicated loci (genes or QTL, for Quantitative Trait Loci), located at homoeologous positions, in the control of agronomic traits. Examples were reported in hexaploid wheat for a large number of traits (flowering time, glutenin synthesis and resistance to cyst nematodes) [5–7] and in soybean for flowering time [8]. Such duplicated homoeologous QTL may often represent a large proportion of the genetic factors controlling a complex trait. In cotton, a meta-analysis of the QTL involved in lint fibre quality showed that 21% of the QTL controlling the trait are located at homoeologous positions [9]. Another recent example of genetic analysis of fruit quality in strawberry revealed that 23% of the QTL detected are homoeologous [10].

*Brassica napus* is a suitable model for studying the effects of WGD on genetic factors involved in the control of complex traits. *B. napus* (2n = 4x = 38, genome: AACC) is an allotetraploid species formed from the hybridization between *B. rapa* (2n = 2x = 20, A genome) and *B. oleracea* (2n = 2x = 18, C genome) [11]. *Brassica* ancestors have undergone two duplication events (α and β) [12, 13] and two triplication events, one ancient event shared by a large majority of Angiosperms (γ) and a more recent event specific to the *Brassica* clade [14]. These WGD events, along with the merger of the two parental genomes, have resulted in a large number of duplicated regions in the *B. napus* genome. Information about the homoeologous relationships between the two genomes of *B. napus* is available. Indeed, Parkin *et al*. [15] identified regions of homoeology through the analysis of intra- and intergenomic duplications in the A and C genomes of *B. napus.* Moreover, the structural organisation of these duplicated genomic regions was studied by taking advantage of the close relatedness of *Brassica sp.* and the model plant *Arabidopsis thaliana* [16, 17]. Twenty four conserved blocks of colinearity were identified between the *Arabidopsis* and *Brassica* genomes [17, 18] in comparative mapping studies which showed that these blocks were highly duplicated in the *B. napus* genome [17, 19]. These duplications are regions that are either orthologous between (located on homoeologous linkage groups) or paralogous within the A and C genomes. As the *B. rapa* and *B. oleracea* genomes contain three subgenomes resulting from a meso-triplication [14], paralogous regions within the *B. napus* A and C genomes could also correspond to ancient homoeologous regions in the *B. rapa* and *B. oleracea* genomes. For this reason, in the present study all these duplicated regions will be referred to as homoeologous/duplicated regions.

Several genetic analyses for various complex agronomic traits such as oil content, seed yield, flowering time [20, 21] and disease resistance [22, 23] have been carried out in *B. napus*, mostly using linkage mapping approaches. Few studies reported duplicated QTL. For example, the involvement of duplicated QTL at homoeologous positions was described for seed glucosinolates [24], flowering time [25], yield-related traits [26] and resistance to sclerotinia stem rot [27]. Due to advances in genome sequencing, comparative mapping and computational technologies, it is now possible to further characterise the duplicated regions involved in the control of complex traits by more precise mapping of QTL and better estimate the proportion of QTL at homoeologous positions. In the present study, we focused on the quantitative resistance to stem canker, caused by the fungal pathogen *Leptosphaeria maculans*, which is one of the major diseases of *B. napus* crops worldwide [28, 29]. Linkage mapping approaches have been used to identify resistance QTL in *B. napus* segregating populations [30–33] and to date, more than twenty QTL have been reported [33, 34]. In our laboratory, QTL detected with linkage mapping studies in bi-parental populations and in one connected population were analysed in relation to the homoeology information provided by Parkin *et al.* [15, 16]. This preliminary analysis suggested that several QTL for resistance to stem canker are localised in homoeologous regions, in particular on linkage groups A1/C1, A2/C2 and A3/C3 (unpublished data). The fact that some QTL are located at homoeologous positions can reflect functional redundancy or be involved in increased allelic diversity of the genes controlling the trait [1, 2, 35, 36]. Knowledge of the resulting diversity in the regions involved in stem canker resistance would help to construct resistant varieties with improved durability with the hypothesis that increasing the diversity of genetic factors controlling the resistance would result in an increase of the potential durability of the resistance [34]. A first step would be to increase the accuracy of detection of homoeologous duplicated QTL using new methods and precise data on the location of duplicated regions in the *B. napus* genome.

The aim of the present study was to assess the proportion of resistance QTL to stem canker located at homoeologous duplicated positions in the *B. napus* genome. To identify genetic regions involved in the resistance, we carried out a genome-wide association analysis. This approach allows the larger genetic diversity present in collections of varieties, compared to bi-parental or connected populations derived from a few parental lines, to be taken into account. Thus QTL detection is more exhaustive than in linkage analysis. The genomic position of the resistance-associated markers was then analysed in relation to the structural organisation of the duplicated regions in the *B. napus* genome in order to estimate the proportion of homoeologous duplicated QTL.

## Results

### Single Nucleotide Polymorphism marker analysis and genetic diversity

The panel of oilseed rape (OSR) varieties was first genotyped with 4329 single nucleotide polymorphism (SNP) markers. Rare alleles were eliminated and a set of 3228 SNP markers with a major allele frequency (MajAF) less than 0.95 was retained. These 3228 markers were well distributed on the *B. napus* linkage map with an average of one SNP every 0.62 centimorgan (cM). A total of 1986 SNP markers was located on the A genome (1 SNP each 0.50 cM) and 1242 SNPs on the C genome (1 SNP each 0.83 cM). The mean polymorphism information content (PIC) value was 0.27 and 0.28 on the A and C genomes, respectively. Out of the 3228 SNP markers, more than 87% showed less than 5% of heterozygous genotyping data and 0.4% had 10 to 13% of heterozygous genotyping data.

### Linkage disequilibrium

The linkage disequilibrium (LD) was evaluated by calculating the *r*^2^ coefficient. Genotyping data of the 3228 SNP markers with a MajAF < 0.95 were used to calculate the *r*^2^ coefficient for the entire panel of 116 OSR varieties. Pairs of markers located on the same linkage group (LG) were defined as linked markers and those located on different LGs were defined as unlinked markers. The mean *r*^2^ was equal to 0.023 for the whole genome. For linked and unlinked markers, the mean *r*^*2*^ was equal to 0.078 and 0.020, respectively. A total of 8.62% and 0.3% of the linked and unlinked tested pairs of SNPs had a *r*^2^ coefficient superior to 0.2, respectively. The percentage of pairs of markers in LD (*r*^*2*^ > 0.2) was 7.77% on the A genome and of 9.57% on the C genome. However, the LD decayed a bit more rapidly on the C genome (up to 1.11 cM) compared with the A genome (up to 1.36 cM) (Additional file 1). On the whole genome, the LD extended up to 1.28 cM, with some variation depending on the LG and the region of the LG considered (Additional file 1, Additional file 2). Overall, markers in strong LD were mapped close to each others on the LGs. However, some exceptions were observed on LG A2, A8 and C8 (Additional file 3) on which LD extended further.

### Population structure and kinship

A set of 727 SNP markers out of the 3228 available was used for Principal Component Analysis (PCA). These 727 SNPs were selected by eliminating close markers that were in strong LD to limit bias in the structure analysis. Results of the PCA (Additional file 4) did not show a strong structure within the population. The variance on the principal eigenvector was mainly due to the accession “Yudal” located at the extreme right of the vector, which is a spring type OSR variety. The two first principal components explained 13.9% of the variation in the panel. Sixteen significant axes that explained 54.7% of the variation within the panel were retained in the Tracy-Widom test. The matrix of coordinates of the accessions on these 16 axes was used as the P matrix for the association analysis.

The distribution of the kinship coefficients, calculated as the proportion of shared alleles between pairs of varieties, is shown in Additional file 5. The mean kinship coefficient value was 0.64. Up to 82% of the pairs of varieties tested had a kinship coefficient value gathered around the mean kinship value, *i.e.* between 0.55 and 0.70. Only a few coefficients showed extreme values. The matrix of kinship coefficients between pairs of varieties was used as the K matrix for the association analysis.

### Marker-trait association analysis

**Figure 1:**
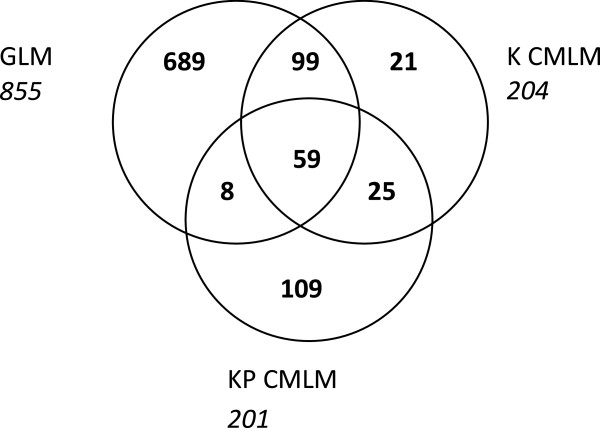
**Number of resistance-associated markers identified with three different linear models.** A panel of 116 winter oilseed rape varieties was used for marker-resistance association analysis. The General Linear Model is a simple linear model, the K Compressed Mixed Linear Model (CMLM) includes the relatedness between individuals of the panel and the KP CML model includes the relatedness and structure of the panel. The number in italics below the model name indicates the total number of markers identified with that model. Marker-trait associations were identified with a type I error of 0.05.

Marker-trait association analysis was carried out with the 3228 SNPs (MajAF < 0.95) on the collection of 116 OSR varieties. Three linear models were used to test the marker/trait associations: (i) a General Linear Model (GLM), which does not take into account the relatedness between individuals, or the population structure; (ii) a K Compressed Mixed Linear Model (K CMLM), which only takes into account the relatedness between the varieties by using the K matrix; (iii) and a KP CMLM, which takes into account both the relatedness and the population structure, by using both the K and P matrices. A total of 1009 markers associated with resistance to stem canker was identified with the three models (type I error α = 5%). Of these 1009 markers, 192 (19%) were identified with at least two models, 689 (68%) were identified with the GLM only, 108 (11%) with the KP CMLM only and 20 (2%) with the K CMLM only (Figure 1). The markers identified with the GLM had overall lower p values than those identified with the K or KP CMLM. Indeed, the p value for approximately 50% of the markers identified with the GLM model was less than 0.01 whereas only 17% and 20% of the markers identified with the KP and K CMLM, respectively, had a p value less than 0.01. When a FDR test was applied at 0.25, no significant associations (α = 5%) were calculated with the K and KP models, whereas 300 (35% of the 855) associations remained significant with the GLM. Of these, 112 were common to the K and/or KP CML models and the remaining 188 markers were located close to markers associated with the K and or KP CML models.

**Figure 2:**
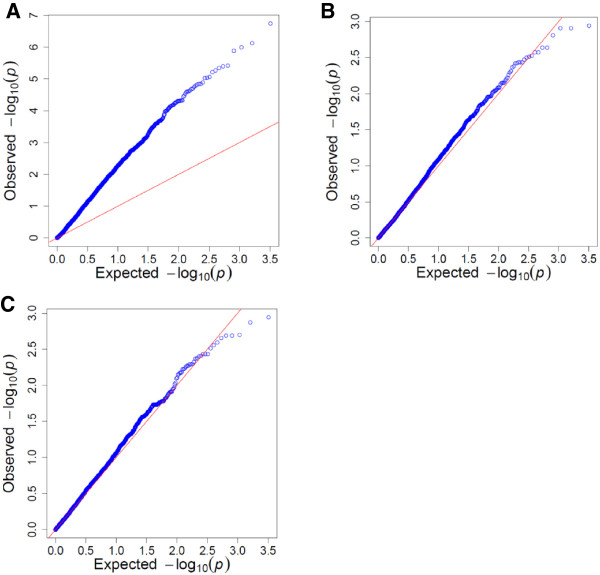
**Regression of the expected –log**_**10**_ **(p value) on the observed –log**_**10**_ **(p value).** A Q-Q plot was used to compare the distribution of observed associations (blue dots) with statistics expected under the null hypothesis of no associations (represented by the red line) for the three linear models: a General Linear model **(A)**, K Compressed Mixed Linear model **(B)** and a KP Compressed Mixed Linear model **(C)**. The negative log_10_(p) was used instead of the p value so that the most significant markers are located at the top right corner of the graphs. A distribution close to the null hypothesis for most of the markers indicated a good control for false positives since it is expected that only a small number of tested markers are truly associated with the resistance.

QQ plots of the observed p-values against the expected p-values under the null hypothesis obtained for the three models suggested that false-positive associations were more likely to be identified with the GLM than with the K and KP models (Figure 2). For the GLM (Figure 2A), deviation from the null hypothesis across the entire distribution indicated bias due to population stratification and/or relatedness [37, 38]. When the relatedness and structure in the panel were included in the model (Figure 2B and C), the deviation from the null hypothesis was corrected, indicating improved control of this type I error.

**Figure 3:**
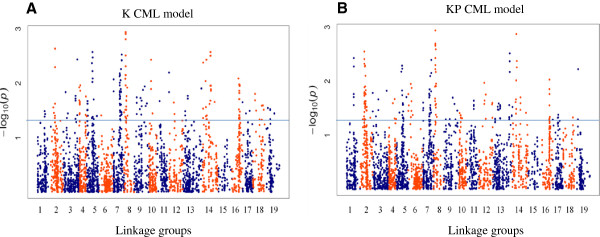
**Genome-wide association analysis of resistance to stem canker in a population of 116 oilseed rape cultivars.** The K Compressed Mixed Linear model **(A)** takes into account the kinship between the varieties while the KP Compressed Mixed Linear model **(B)** takes into account the kinship and the structure of the varieties within the panel. Negative log_10_ of p-values were plotted against the genetic distance in cM on the 19 *B. napus* linkage groups (from 1 to 10: LGs A01 to A10; from 11 to 19: LGs C01 to C09). The horizontal line indicates the genome-wide association significance threshold.

Because of their capacity to control false-positive associations, in further analyses we focused on results from the K and KP models. The K and KP CML models identified a total of 321 markers (Additional file 6) significantly associated with resistance to stem canker (p-value < 0.05) (Figure 3). Eighty four markers (26%) were identified with both models, 120 (37%) with the K CMLM only and 117 (36%) with the KP CMLM only. The associated markers are located on all LGs, except for the LG C5 where no association was identified (Figure 3). Among these resistance associated markers, 217 markers are located on the A genome and 104 markers on the C genome. This represents 10.9% of the 1986 markers mapped to the A genome and 8.4% of the 1242 markers mapped to the C genome.

### Structural organisation of the markers associated with resistance to stem canker

**Figure 4:**
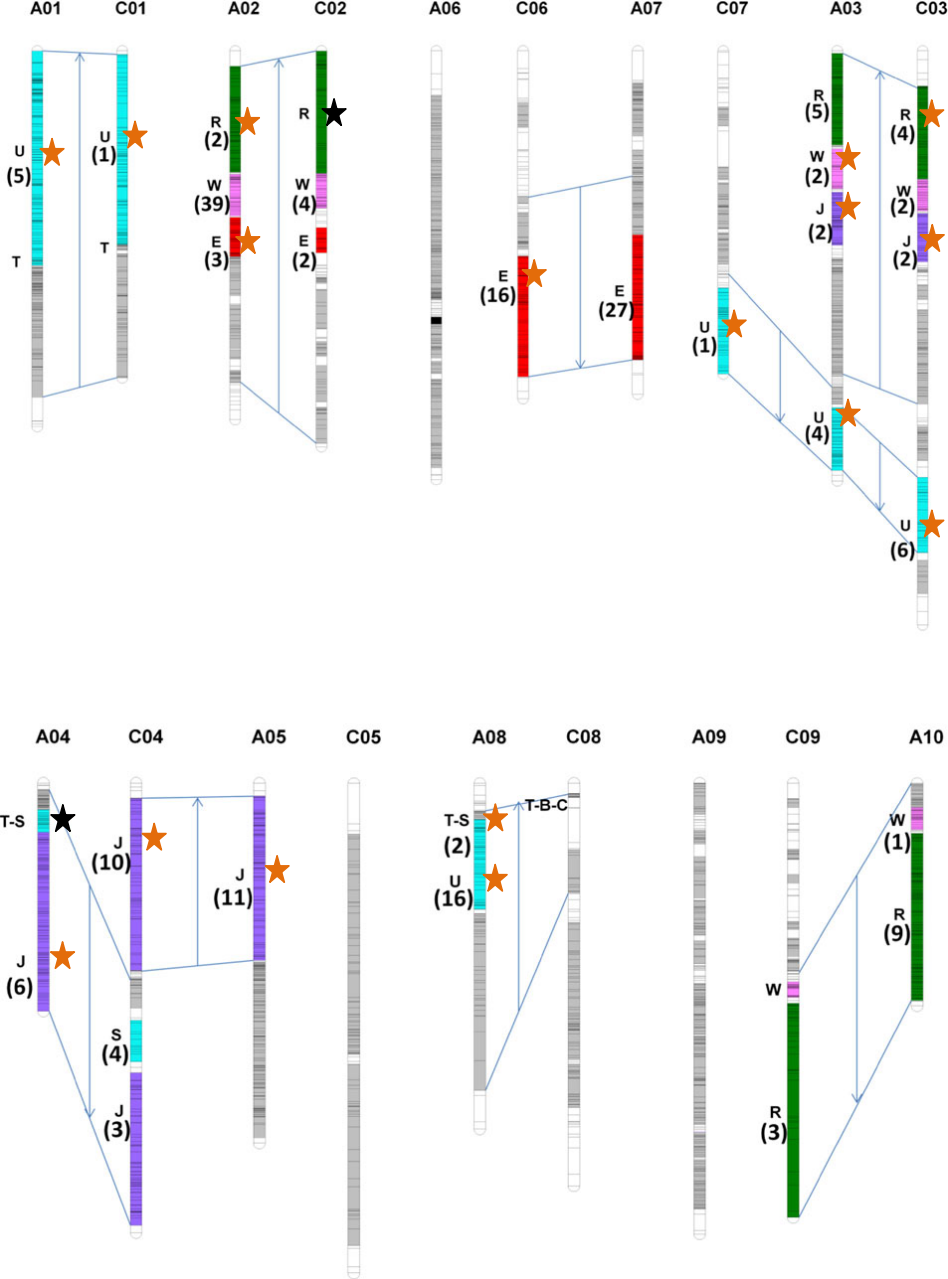
**Distribution of resistance-associated markers located in strictly duplicated regions of the** ***B. napus*** **genome.** The coloured segments correspond to the six duplicated blocks, as defined by Schranz et al. [17], on which associated markers were identified in regions collinear to the same *A. thaliana* sequence interval. All the known duplications of these six blocks in the *B. napus* genome are shown. The number of resistance-associated markers identified on each block is indicated in parenthesis. The absence of a number indicates that no association was identified in the region. The resistance QTL identified in bi-parental or connected populations by linkage mapping on the copies of the 6 duplicated blocks are represented by stars on the right of the linkage groups. Orange stars indicate co-localisation of associated markers and previously identified QTL, black stars indicate loci where QTL were detected but where no resistance associated marker was identified. Linkage groups are organized according to the colinearity between the A and C genomes. The orientation of the arrows indicates the orientation of the colinearity between the genomic regions.

The structural organisation of the resistance-associated markers was then analysed in relation to their location on the 24 conserved blocks of colinearity between *B. napus* and *A. thaliana* (henceforth named AK blocks) and to their anchorage on *A. thaliana* genes. The results are presented in Table 1 and in Additional file 7. Out of the 321 associated markers, 279 were assigned to one unique block, 25 to two or three different blocks and 17 could not be assigned (Additional file 6). The 279 markers with unique anchorage were located on 23 AK blocks corresponding to 64 distinct genomic regions (Additional file 7). For nine of these genomic regions, resistance associated markers were identified only on one copy of the corresponding nine blocks. For the other 55 genomic regions, resistance associated markers were identified on at least two copies of the 14 corresponding blocks. This suggests that more than 85% (55 out of 64) of the genomic regions associated with resistance to stem canker are duplicated regions. A more detailed analysis, based on the correspondence between resistance associated markers and *A. thaliana* genes, was carried out to investigate these 55 duplicated regions. In 28 genomic regions, corresponding to six of the 14 blocks, resistance-associated markers had significant hit against the same interval of the *A.thaliana* sequence suggesting that these are located in strictly duplicated regions (Table 2). In the other 27 regions corresponding to the eight other blocks, resistance-associated markers aligned with neighbouring intervals of the *A. thaliana* sequence suggesting that these resistance-associated are not strictly located in the same duplicated region (Additional file 7). We then examined more closely the resistance-associated markers in the six strictly duplicated regions on the E, J, R, T U and W blocks. Within the *B. napus* genome, the E block is duplicated four times, and the J, R, T, U and W blocks are duplicated six times (Figure 4). Resistance-associated markers were identified on the four duplicated E blocks, on the six duplicated J and U blocks, on two of the six duplicated T blocks and on five of the six duplicated R and W blocks. Thus, out of the 34 genomic regions corresponding to the duplication of the six investigated blocks, 28 (82%) carried resistance-associated markers. These 28 genomic regions grouped 69% of the 279 associated markers assigned to one unique AK block and 60% of the total 321 associated markers identified. Details of the organisation of the 28 regions with resistance associated markers on the *B. napus* genome are shown in Figure 4. Twenty-three of the 28 genomic regions were located on five collinear homoeologous LGs: A2/C2, A3/C3, A5/C4, A7/C6 and A10/C9. In four of the 28 genomic regions, QTL were identified on only one of the two homoeologous LGs. Finally, one of the 28 QTL (on block U, LG A8) was located in a region for which no homoeologous region has been identified in the *B. napus* genome to date. The number of resistance-associated markers detected and their location in the six duplicated blocks of interest are presented in Table 2.

**Table 1:** **Distribution of genomic regions associated to stem canker resistance in relation to the organisation of duplicated blocks in the** ***B. napus*** **genome**

	Regions with resistance associated markers	Regions with resistance associated markers on only one copy of a block	Regions with resistance associated markers on at least two copies of a block		
			Total	Same duplicated regions	Neighbouring duplicated regions
Number of blocks	23	9	14	6	8
Number of genomic regions with resistance associated markers	64	9	55	28	27
Total number of duplications of the blocks in *B. napus* genome	124	40	84	34	50
Percentage of duplications with resistance associated markers	51.6	22.5	65.5	82.3	54.0

**Table 2:** **Resistance-associated markers detected in strictly duplicated regions**

AK block	LG	SNP markers		Interval size (cM)	Marker density in the interval	Linkage disequilibrium		A. ***thaliana***genes interval corresponding to the ***B. napus***region with RAM	Co-localisation of RAM with previously detected QTL (number of RAM)
		Total SNP	Number of RAM			Mean r ^2^	Percentage of r ^2^ value > 0.2		
E	A2	53	3 *(6)*	7.90	0.15	0.28	40.13	AT1G65980-AT1G70790	3
	A7	97	27 *(28)*	40.00	0.41	0.10	13.57	AT1G67300-AT1G80680	0
	C2	17	2 *(12)*	4.70	0.28	0.53	51.37	AT1G68370-AT1G68410	0
	C6	92	16 *(17)*	37.90	0.41	0.24	37.11	AT1G67230-AT1G74920	12
J	A3	29	2 *(7)*	9.60	0.33	0.18	29.56	AT2G33830-AT2G35680	2
	A4	55	6 *(11)*	28.90	0.53	0.11	15.63	AT2G32810-AT2G43230 and AT3G58500.1	1
	A5	97	11 *(11)*	46.20	0.48	0.11	16.07	AT2G32650-AT2G42680	4
	C3	49	2 *(4)*	12.70	0.26	0.14	19.98	AT2G38570	2
	C4a	45	10 *(22)*	50.80	1.13	0.08	9.19	AT2G36490-AT2G46680.1	9
	C4b	34	3 *(9)*	24.80	0.73	0.16	28.70	AT2G35500-AT2G40570	0
R	A2	27	2 *(7)*	26.70	0.99	0.11	10.83	AT5G17770-AT5G19360	2
	A3	53	5 *(9)*	29.30	0.55	0.20	29.68	AT5G02050.1-AT5G15150 and AT5G20890	0
	A10	92	9 *(10)*	47.40	0.52	0.10	13.90	AT5G15950-AT5G20970	0
	C3	46	4 *(9)*	29.40	0.64	0.13	21.37	AT5G17790-AT5G18960	1
	C9	20	3 *(15)*	61.70	3.09	0.11	11.58	AT5G13850-AT5G13870	0
T	A8	5	2 *(40)*	2.20	0.55	0.73	100.00	AT4G14230.1-AT4G14320	1
	C4	4	4 *(100)*	0.80	0.20	0.53	100.00	AT4G13780-AT5G14330	0
U	A1	80	5 *(6)*	60.30	0.75	0.07	8.58	AT4G28230-AT4G28380	5
	A3	64	4 *(6)*	20.10	0.31	0.09	10.66	AT4G18050-AT4G18197	2
	A8	36	16 *(44)*	22.90	0.64	0.21	35.89	AT4G25610-AT4G34135	16
	C1	60	1 *(2)*	60.70	1.01	0.09	10.01	AT4G33330	1
	C3	25	6 *(24)*	24.10	0.96	0.23	33.54	AT4G20390-AT4G34450	4
	C7	54	1 *(2)*	26.70	0.49	0.09	13.07	AT4G18050.1	1
W	A2	65	39 *(60)*	14.80	0.23	0.29	52.77	AT5G49810-AT5G59840	0
	A3	17	2 *(12)*	12.80	0.75	0.20	28.68	AT5G53140-AT5G60220	2
	A10	29	1 *(3)*	5.10	0.18	0.18	27.59	AT5G53530	0
	C2	21	4 *(19)*	10.00	0.48	0.14	23.33	AT5G54690-AT5G57580	0
	C3	43	2 *(5)*	10.00	0.23	0.20	34.44	AT5G50920	0

For each duplicated regions on which resistance-associated markers (RAM) were identified, information is provided about the number and proportion (in parentheses) of resistance-associated markers, the size of the intervals with RAM, the marker density and the linkage disequilibrium in the interval. The correspondence of resistance-associated markers with *A. thaliana* gene intervals is also shown. The co-localisation of resistance-associated markers with previously identified QTL (in a double haploid and/or a connected population also from our laboratory) is indicated.

## Discussion

This study highlighted that a large proportion of genomic regions involved in resistance to stem canker is located in duplicated homoeologous regions of the *B. napus* genome. These results were obtained using a genome-wide association analysis with a large number of SNPs on a panel of oilseed rape (OSR) varieties and by exploiting information available on the structural organisation of duplicated blocks in the *B. napus* genome.

### Genome-wide association studies (GWAS) allow the identification of QTL in polyploid species

Advances in genome sequencing and computational technologies have led to high throughput SNP discovery in polyploid species [39] including *B. napus* [40]. Thus a large number of markers mapping at single loci are now available that can be used to conduct a precise GWAS as in diploid species. The power of association detection by GWAS partly depends on marker density and the extent of LD [41]. In the present study the average genome wide LD decayed within 1.28 cM, supporting previous studies in winter oilseed rape collections [40, 42, 43]. The average marker density (1 SNP every 0.62 cM) and the extent of LD in our panel allowed high-power detection of resistance-associated markers over the whole genome. Two LGs where the extent of LD was less than the marker density were the exception (Additional file 1). We limited the detection of false positive associations by taking into account the population structure and the varieties relatedness as previously reported [44]. Results from the QQ-plots showed that the K and KP models were better at limiting the false positive association rate than the GLM. This conclusion has been made in several other association mapping studies which also took into account the panel structure [44, 45]**,** including that carried out by Jestin et al. [46] on the expanded OSR panel.

Some of the resistance-associated markers identified here are localised in genomic regions where we previously detected QTL by linkage mapping. A total of 28% of the associated markers identified with the K and KP CML models co-localised with 55% and 78% of the QTL detected in the biparental DYDH segregating population and in a connected multiparental population, respectively [30, 31, 47]. Some other regions were detected with only one or the other method. Differences between genomic regions identified by association mapping and linkage analysis have been observed in *B. napus* for other traits such as oil content [48] as well as in other species [8]. These differences are due to the type of population used, the allele frequency in the population, the type and number of markers used or the genetic map coverage. Our results show that a large proportion of QTL identified in the segregating populations can be detected in the association panel but also that new sources of resistance are present in the panel. This highlights the need to use both QTL detection methods in combination to exhaustively dissect the genetic architecture of complex traits for plant breeding.

In this study, we focused on the quantitative resistance to stem canker. Qualitative resistance controlled by specific major resistance genes have also been identified in *B. napus* [34]. These genes are *Rlm2* on LG A10 and *Rlm1* and the putative cluster *Rlm3-Rlm4-Rlm7-Rlm9* on LG A07 [34]. To maximise chances to only study the quantitative resistance, the OSR varieties in our panel were chosen because they did not carry any effective specific resistance gene to *L. maculans* in our field conditions. Thus, we excluded OSR varieties known as carrying *Rlm7,* a highly effective gene that was recently introduced in OSR varieties in France. However, we cannot exclude that some associated markers might be linked to genes with a lower level of effectiveness. Indeed, associated markers located on the E bloc on linkage group A07 are in the region of the *Rlm1* gene indicating that these markers could be linked to this specific resistance gene. In contrast, no associated markers were detected in the vicinity of the *Rlm* cluster region on A07.

### Organisation of QTL for resistance to stem canker in the *B. napus*genome

All of the resistance associated markers identified by GWAS corresponded to 64 genomic regions involved in the control of stem canker. These genomic regions are quite equally distributed on the A and C genomes of *B. napus* indicating that both genomes contribute to stem canker quantitative resistance. The number of genomic regions involved in resistance identified in this study is higher than that found by Jestin et al. [46] in the expanded panel of WOSR, mainly due to the use of a denser map. By exploiting the collinearity between the *A. thaliana* and *B. napus* genomes, we found that 28 (44%) of the 64 regions associated to resistance correspond to strictly duplicated genomic regions. Some other genomic associated regions correspond to neighbouring duplicated genomic regions of some of the AK blocks. If the accuracy of the GWAS analysis can be improved, we may find that some of these regions also correspond to strictly duplicated genomic regions. Thus it appears that more than 44% of the genomic regions involved in resistance to stem canker are strictly duplicated. This percentage of homoeologous genomic regions controlling a complex trait is higher than that reported so far in other polyploid species. Indeed, in allotetraploid cotton, Rong *et al*. [9] estimated that 21% of the 196 QTL involved in the control of cotton lint fibre quality are homoeologous QTL. In allooctoploid strawberry, Lerceteau-Kholer *et al*. [10], found that 23% of the 87 QTL involved in controlling strawberry fruit quality are homoeologous QTL. It is obvious that the proportion of homoeologous duplicated QTL that can be identified depends on the ability to exhaustively detect causative variants and the accuracy in identifying homoeologous regions (which requires a complete dissection of the genome structure). In addition, the proportion of homoeologous duplicated QTLs that can be observed also depends on the divergence time between the parental species and the evolutionary process (mainly artificial or natural selection) taking place in the history of these species for the studied trait. Thus it is difficult to conclude if there are really more duplicated genomic regions involved in the control of a quantitative trait in *B. napus* as compared with other polyploid species.

However, the high percentage of identified homoeologous genomic regions involved in resistance to stem canker in *B. napus* could be tightly linked to the significant structural conservation between *B. rapa*, *B. oleracea* and the A and C genomes of *B. napus* [49]. In addition, the two parental species have diverged more recently compared to other polyploid species such as cotton [14, 50]. Li et al. [51] recently identified QTL for morphological and yield component traits in duplicated regions within the A genome of *B. rapa* and in homoeologous regions between the A, B, and C genomes of *B. rapa*, *B. juncea* and *B. napus*, which supports the strong level of conservation between Brassica genomes.

Another factor affecting the relatively large proportion of duplicated homoeologous genomic regions involved in resistance to stem canker may be the nature of the trait. It is assumed that polyploidization events result in redundancy of duplicated genes but this gene redundancy can be reduced in a long term evolutionary process through various diploidization mechanisms among which genome fractionation is a major force [2, 3, 52]. Fractionation has been shown in polyploid species such as Arabidopsis [53], maize [54, 55] or *B. rapa* [56]. However, various studies highlighted that some functional gene categories are preferentially preserved from this reduction and are over-retained in duplicated copies. These include transcriptional factors, and protein kinases in the Arabidopsis lineage [57, 58], genes involved in signal transduction and some responses to external stimuli in rice [59], and genes involved in networks with a high level of connectivity in soybean [60]. In *B. rapa*, a recent study showed that genes involved in resistance to pathogens and especially in broad spectrum defence, were over-retained after the ancestral triplication event [61]. Given that the genomes of *B. napus* and *B. rapa* are highly conserved, it can be hypothesized that the large number of identified genomic regions involved in resistance to stem canker is tightly linked to this over-retention phenomenon. However, some genome fractionation or modification of gene expression/regulation could have occurred in some of the duplicated regions which would explain why, in some cases, resistance associated markers or QTL were not found on all the homoeologous copies in *B. napus* genome. Indeed, small-scale deletions have been observed at the genome microstructure level and in sequences in Brassica [49]. Examples of gene neofunctionalisation and subfunctionalisation have also been observed in *B. rapa* [62–64]. These modifications were also extensively demonstrated in synthetic *B. napus* allotetraploids after the polyploidization process [65, 66] and in stabilized natural *B. napus* [49]. Nevertheless, the absence of resistance associated markers at some homoeologous loci could also be due to our experimental set-up. The causative variants at the “missing” QTL may be too rare, or even absent, in the panel to be detected through association mapping. Indeed, some of these “missing” QTL were identified in previous studies by linkage mapping, which is a more efficient method for detecting rare alleles [30, 46, 47].

It will now be interesting to further investigate the regions carrying the homoeologous QTL identified in this study. Characterisation of their gene content and expression, and sequence similarities will help elucidate the consequences of their conservation and evolution on the diversity of genetic factors involved in quantitative resistance to stem canker.

## Conclusions

Our GWA study showed that many homoeologous duplicated regions are involved in the control of resistance of *B. napus* to stem canker. The resistance related genomic regions identified with GWAS overlapped with those identified by linkage mapping but this study also provided new information. Thus both methods can be valuable for a complete dissection of the architecture of genomic regions controlling complex traits. Our results showed that both the A and C genomes equally contribute to stem canker resistance and that 44% of the regions involved in the control of the resistance corresponded to homoeologous duplicated regions. In these regions, the associated markers were located in strictly duplicated intervals of six blocks which are syntenic with *A. thaliana*. Further studies to characterise the similarity/divergence in gene content and sequence of these duplicated regions are needed to gain insight into the conservation and allelic diversity of the underlying genes.

## Methods

### Plant material

A panel consisting of 115 European winter oilseed rape (OSR) varieties and one Asiatic spring OSR variety was used. This panel is a subset of that used in Jestin *et al.* [46]. Most of the varieties in the panel are double low, *i.e.* without erucic acid and with low glucosinolate seed content, and are registered in the French and the European catalogues (Additional file 8). These varieties were chosen because they did not carry any effective specific resistance gene to *L. maculans* in our field conditions. Thus, we excluded OSR varieties known as carrying *Rlm7,* a highly effective gene that was recently introduced in OSR varieties in France *.* Moreover, varieties of the panel showed a large range of responses to the infection, according to previous results obtained in different trials from different years [46].

### Phenotypic evaluation

The material was previously evaluated in a field trial in 2006 at Le Rheu (France) [46]. For the phenotypic evaluation a disease index called the “G2 index” was calculated to classify the genotypes on a scale running from 0 to 9; from the most resistant (G2 index = 0) to the most susceptible varieties (G2 index = 9). The G2 index indicates the area of necrosis at the base stem section of the plant [44]. Statistical analysis of field trial results are presented in Jestin *et al.* [46].

### Statistical analysis of genotyping data

The whole panel was genotyped with 5685 SNP markers. Within these markers, 4329 were mapped on an integrated map generated in our laboratory [40]. The map covers 2027.7 cM, which represents a marker density of 2.56 SNP per cM. A total of 2839 (65.6%) and 1490 (34.4%) of these SNP markers were mapped on the A and the C genome of *B. napus,* respectively. Only the genotyping data obtained with the mapped 4329 SNP markers were conserved for the following analysis.

The major allele frequency (MajAF), percentage of heterozygosity and polymorphic information content (PIC) were estimated for each SNP marker using PowerMarker v3.25 software [67]. Markers with a MajAF ≥ 0.95 and varieties with more than 15% of heterozygous genotyping data were removed from the data set to prevent bias during the association analysis.

### Linkage disequilibrium

LD was evaluated on the whole genome and on each LG by calculating the correlation coefficient *r*^2^ between each pair of markers located on the same LG (linked markers) and for pairs of markers located on different LGs (unlinked markers). This analysis was performed using PLINK v1.07 software [68]. LD decay was evaluated using a nonlinear regression of the expected *r*^2^ as described by Sved [69] with E[r^2^] = 1/(1 + 4N_e_c) where c is the recombination rate in morgans and N_e_ the effective population size. The E[r^2^] was plotted against the genetic distance between molecular markers (in cM) in order to estimate the extend of LD with R software [70]. The *r*^*2*^ threshold value of 0.2 was chosen as the value below which the LD is no longer significant. Heat maps of LD between markers of the same LG were generated using the LDHeatMap package developed on R software [71].

### Population structure and kinship

To investigate population structure, close markers that were in LD were first eliminated because these might cause a bias in the estimation of population structure. PLINK v1.07 [68] was used to select the markers. A sliding window of 20 SNP was defined with a 2-SNP step. In each window, the *r*^2^ coefficient was calculated for each possible marker pair. When the *r*^2^ value was greater than 0.2, one of the markers of the pair was eliminated so that only the markers that are not in LD were retained in the window. The set of markers obtained after this first analysis was then used to lead a principal component analysis (PCA) on the basis of genotyping data using the software EIGENSTRAT [72]. A Tracy-Widom test (alpha = 5%) was applied to identify significant axes of the PCA. The matrix of coordinates of the varieties on these significant axes, called the P matrix, was used to control the population structure in the association analysis.

An identity by state (IBS) kinship matrix was computed as a genotype similarity between the different pairs of markers using the algorithm “EMMA” in the GAPIT package [73]. The matrix of kinship values between pairs of varieties was used as a K matrix in the association analysis.

### Genome-Wide Association analysis

Association mapping analysis was carried out using the GAPIT package [73]. All the markers with a MajAF < 0.95 were used in three different linear models to test the association between molecular markers and the G2 disease index trait. First, the GLM was applied. In this model, each marker is considered as an independent variable and there is no control over the population structure or kinship. Then, the K CMLM was used. In this model, a kinship matrix (K) is used to take into account the relationship between individual genotypes. Finally, the KP CMLM allows both the population kinship and structure to be controlled using the K and P matrices. Compared with a simple mixed linear model, compressed mixed linear models increase the statistical power and reduce computing time for large samples [74]. The K and KP CM linear models should allow better control of false associations caused by population structure.

### Structural organisation of stem canker QTL

To study the organisation of the genetic factors controlling resistance to *L. maculans* in relation to the duplications of *B. napus* genome, our integrated genetic map was anchored on the Arabidopsis genome using the homology with the SNP context sequences [40]. A map with the duplicated blocks on our integrated map was constructed and blocks were presented in relation to the 24 blocks defined by Schranz *et al.* [17] and recently refined by Cheng *et al*. [18].

### Availability of supporting data

The context sequences of the SNP markers mentioned as private in Additional file 6 can be requested from S. Faure (Sebastien.Faure@biogemma.com).

## Electronic supplementary material

Additional file 1: Table S1: Extent of linkage disequilibrium on each linkage group of *B. napus*. Extent of linkage disequilibrium was calculated according to the formula E[r^2^] = 1/(1 + 4N_e_c) as described in Sved *et al*. [69]. The effective population size (Ne) and the distance to which the LD extended (c) at a *r* ^*2*^ threshold of 0.2 are given for each linkage group, for the A and C genomes and for the whole genome. (XLSX 10 KB)

Additional file 2: Figure S1: Linkage disequilibrium related to the genetic distance between markers along *B. napus* genome. The linkage disequilibrium decay was measured in the whole panel of 116 winter oilseed rape cultivars. The red curve shows the non-linear regression trend line of *r* ^*2*^ versus genetic distance. (PDF 125 KB)

Additional file 3: Figure S2: The linkage disequilibrium pattern and results for association analysis of resistance to stem canker for each linkage group. Negative log_10_ p values obtained from the K and KP CML models were plotted against the genetic distance (in cM) for each linkage group. The corresponding linkage disequilibrium pattern calculated between pairs of tested markers for association is presented below the Manhattan plot. The more the colour is closer to red, the higher the linkage disequilibrium is. (PDF 1 MB)

Additional file 4: Figure S3: Principal Component Analysis of 116 winter oilseed rape cultivars based on 727 SNP markers. The proportion of variance explained by the two principal components (Eigenvector 1 and 2) is indicated in parentheses. Varieties were tagged according to their euric acid and glucosinolate contents, “00” indicates a double low in erucic acid and glucosinolate content, “0+” indicated low erucic acid and high glucosinolate content, “+0” indicates high erucic acid and low glucosinolate content and “++” indicated high erucic acid and high glucosinolate content. The spring type variety ‘Yudal’ is circled in red on the right of the graph. (PDF 167 KB)

Additional file 5: Figure S4: Distribution of the kinship coefficients calculated between 116 winter oilseed rape cultivars. (PDF 171 KB)

Additional file 6: Table S2: List of the 321 resistance associated markers. The positions (in cM) of the markers on our linkage map, including information about their assignation to AK block are provided. When possible, context sequences of associated SNP are given. For a set of markers mentioned “private”, context sequences are available under request (see ‘Availability of supporting data’ section). (XLSX 33 KB)

Additional file 7: Table S3: Position of resistance-associated markers in relation to the duplicated blocks in *B. napus* genome. P values given in the table for the K and KP linear models were those not corrected with the FDR test. Information about the co-localisation of resistance-associated markers with previously identify QTL in our laboratory (in a double haploid -DH- and/or a connected -CP- populations) is indicated. (XLSX 20 KB)

Additional file 8: Table S4: List of *Brassica napus* varieties used for the genome-wide association analysis. *The G2 index is a mean of three replicates. **“00” indicates a double low in erucic acid and glucosinolate content, “0+” indicated low erucic acid and high glucosinolate content, “+0” indicates high erucic acid and low glucosinolate content and “++” indicated high erucic acid and high glucosinolate content. The dash indicate missing data. IHAR: Instytut Hodowli I Aklimatyzacji Roslin, Poznan; INRA: Institut National de la Recherche Agronomique; JD: Jouffray-Drillaud; KWS: KWS Saat AG; Momont: Sarl Adrien Momont & Fils; NK: Syngenta Seeds SAS; NPZ: NPZ Lembke Semences Sarl; RAPS Gbr: Raps GbR Saatzucht Lundsgaard; SW: Svalöf Weibull AB. (XLSX 15 KB)
