# Supplementary figures and images for: Homoeologous duplicated regions are involved in quantitative resistance of Brassica napus to stem canker
Source: BMC Genomics. 2014 Jun 19;15(1):498. doi: 10.1186/1471-2164-15-498 (PMC4082613; doi:10.1186/1471-2164-15-498)

Additional file 2-Figure S1

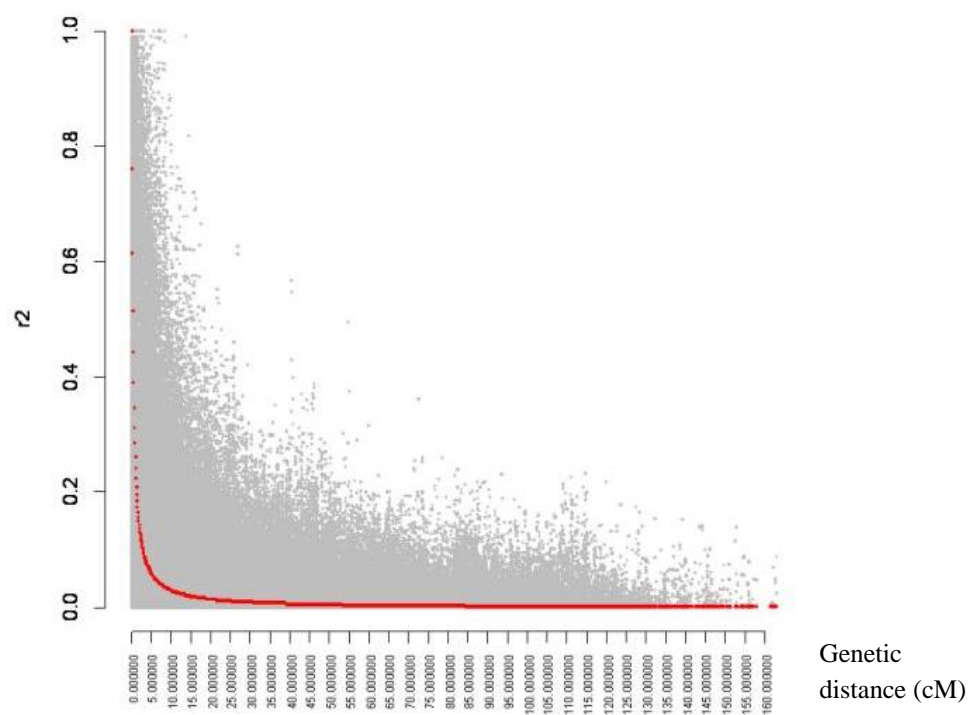

Supplement: Supplementary file 2 — Additional file 2: Figure S1: Linkage disequilibrium related to the genetic distance between markers along B. napus genome. The linkage disequilibrium decay was measured in the whole panel of 116 winter oilseed rape cultivars. The red curve shows the non-linear regression trend line of r 2 versus genetic distance. (PDF 125 KB) [file 12864_2013_6190_MOESM2_ESM.pdf]

Additional file 4-Figure S3

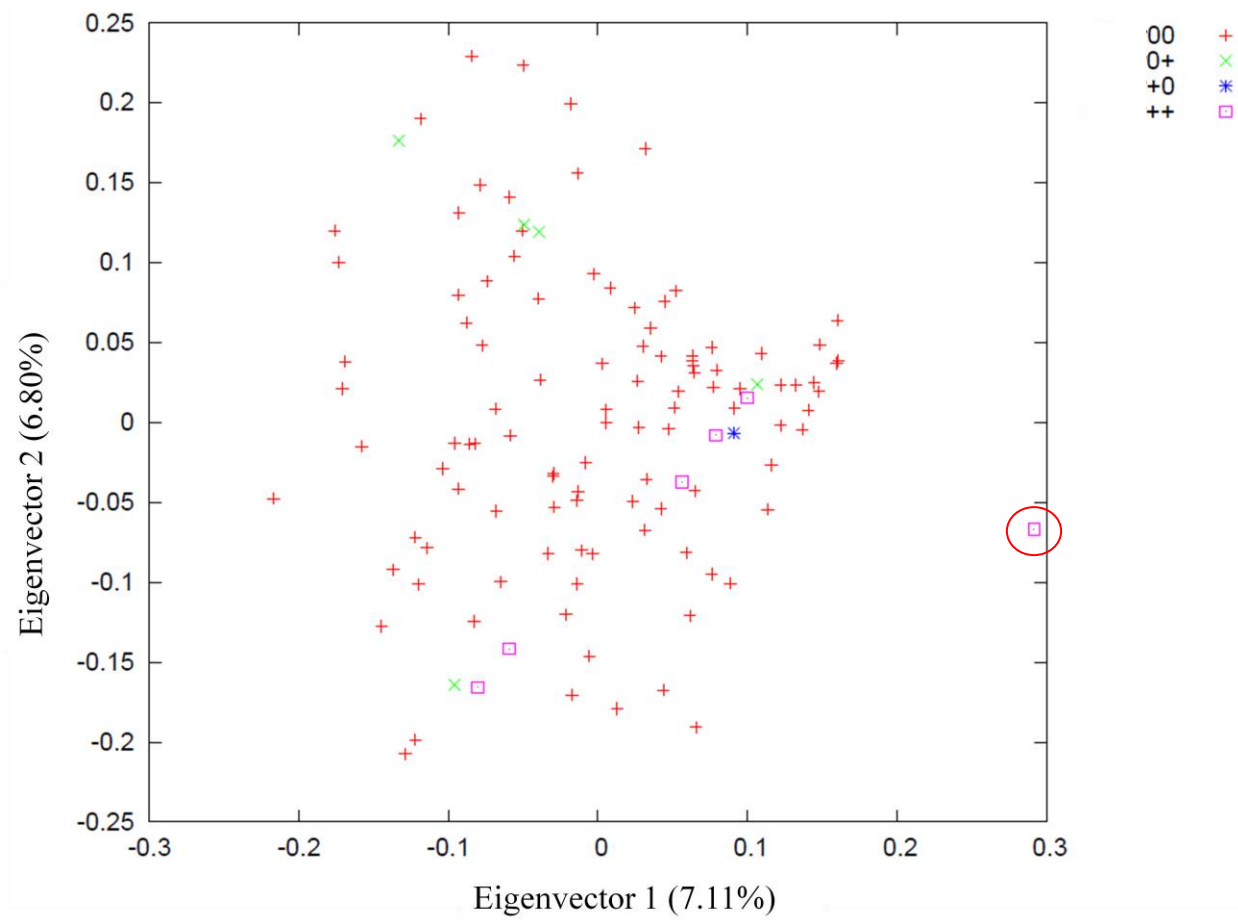

Supplement: Supplementary file 4 — Additional file 4: Figure S3: Principal Component Analysis of 116 winter oilseed rape cultivars based on 727 SNP markers. The proportion of variance explained by the two principal components (Eigenvector 1 and 2) is indicated in parentheses. Varieties were tagged according to their euric acid and glucosinolate contents, “00” indicates a double low in erucic acid and glucosinolate content, “0+” indicated low erucic acid and high glucosinolate content, “+0” indicates high erucic acid and low glucosinolate content and “++” indicated high erucic acid and high glucosinolate content. The spring type variety ‘Yudal’ is circled in red on the right of the graph. (PDF 167 KB) [file 12864_2013_6190_MOESM4_ESM.pdf]

Additional file 5-Figure S4

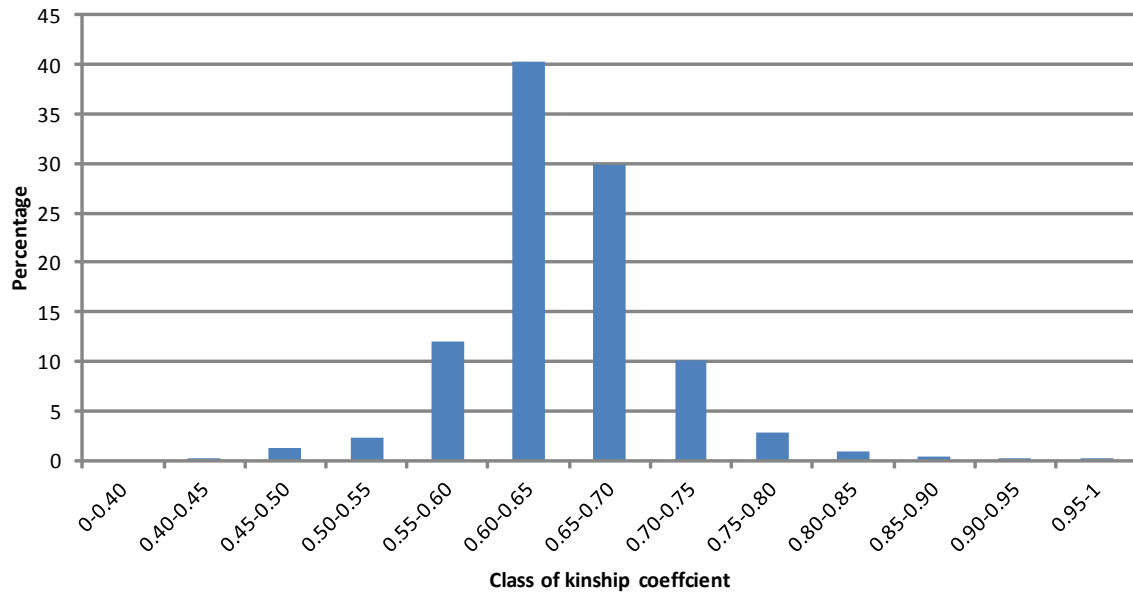

Supplement: Supplementary file 5 — Additional file 5: Figure S4: Distribution of the kinship coefficients calculated between 116 winter oilseed rape cultivars. (PDF 171 KB) [file 12864_2013_6190_MOESM5_ESM.pdf]
