# Supplementary material for: Homoeologous duplicated regions are involved in quantitative resistance of Brassica napus to stem canker
Source: BMC Genomics. 2014 Jun 19;15(1):498. doi: 10.1186/1471-2164-15-498 (PMC4082613; doi:10.1186/1471-2164-15-498)

Additional file 3-Figure S2

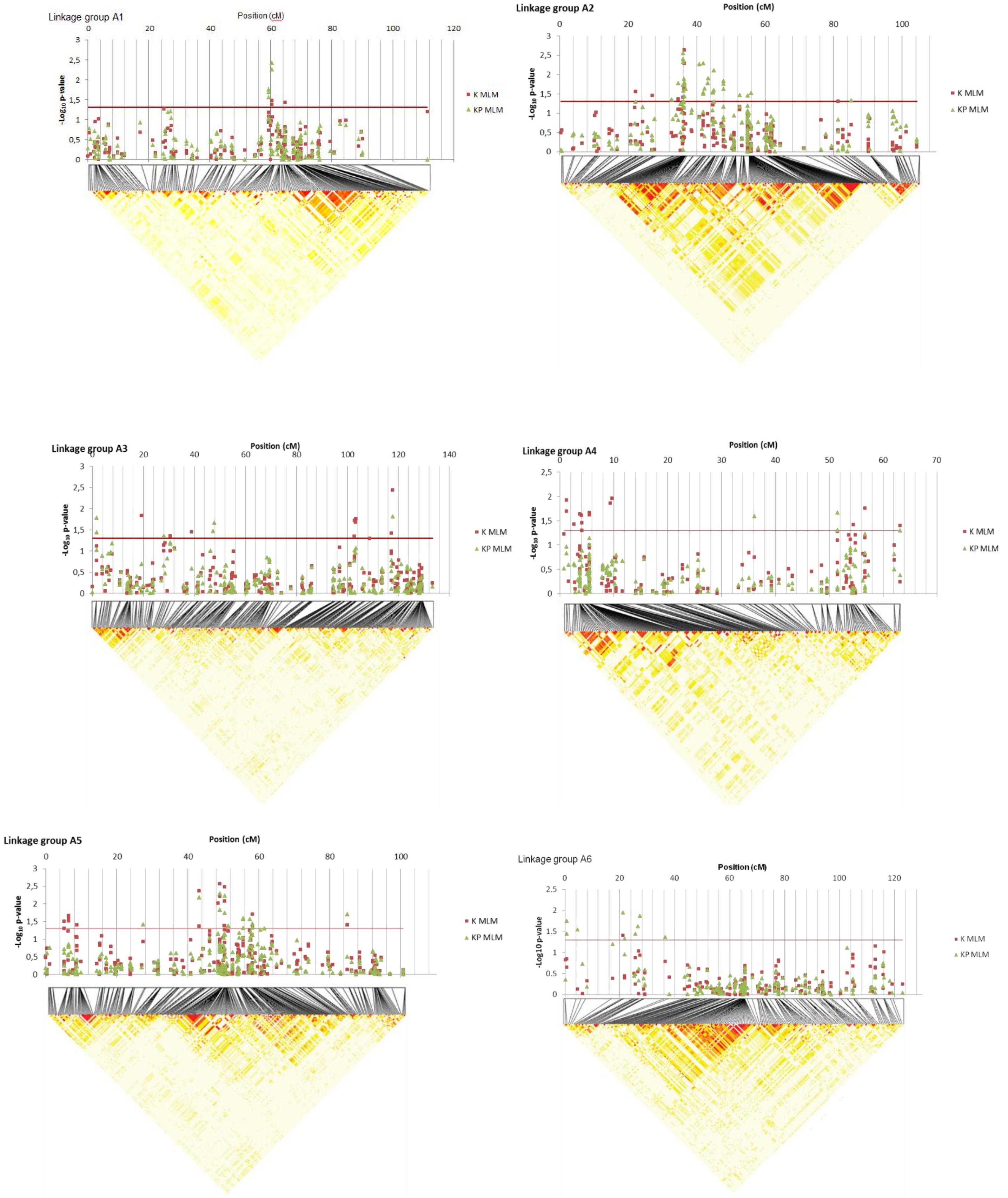

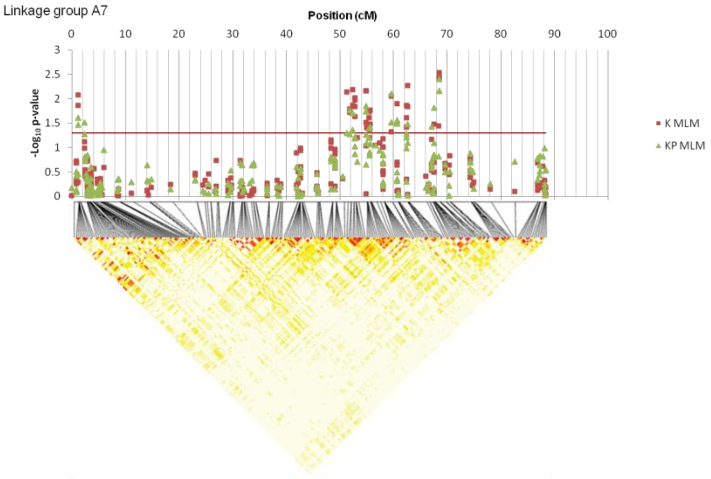
Linkage group A7
Position (cM)
-Log10 p-value
K MLM
KP MLM

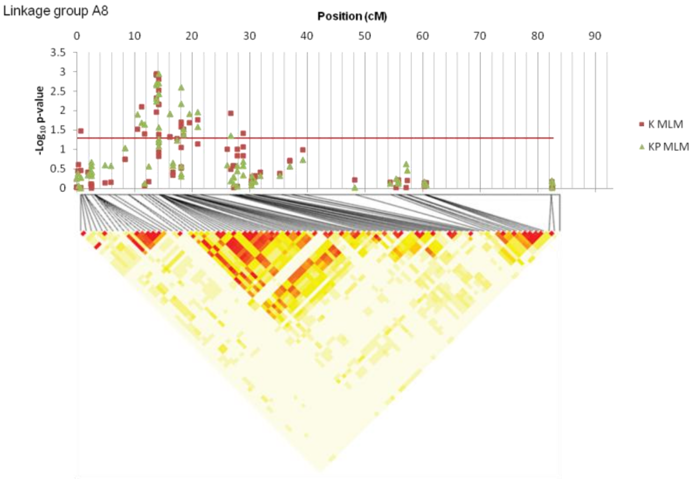
Linkage group A8
Position (cM)
-Log10 p-value
K MLM
KP MLM

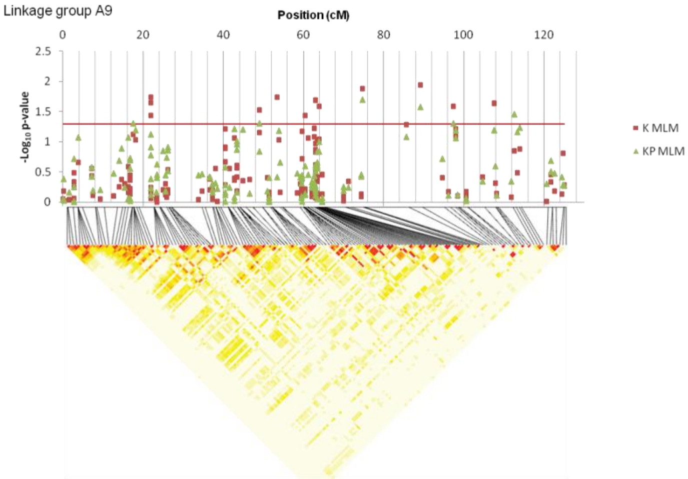
Linkage group A9
Position (cM)
-Log10 p-value
K MLM
KP MLM

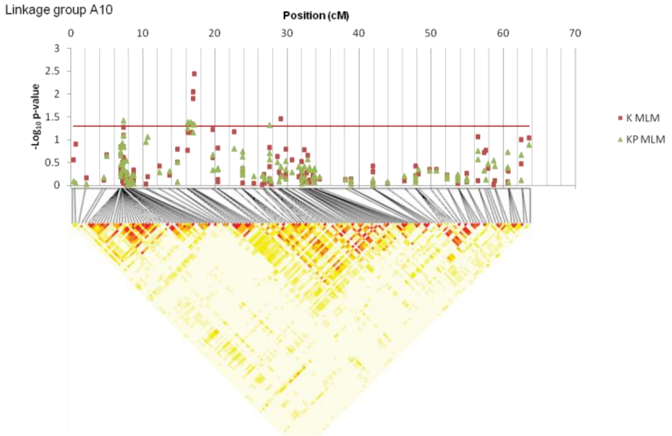
Linkage group A10
Position (cM)
-Log10 p-value
K MLM
KP MLM

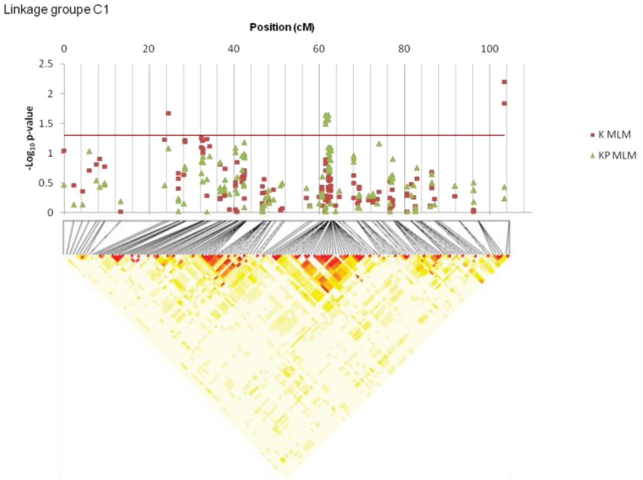
Linkage groupe C1
Position (cM)
-Log10 p-value
K MLM
KP MLM

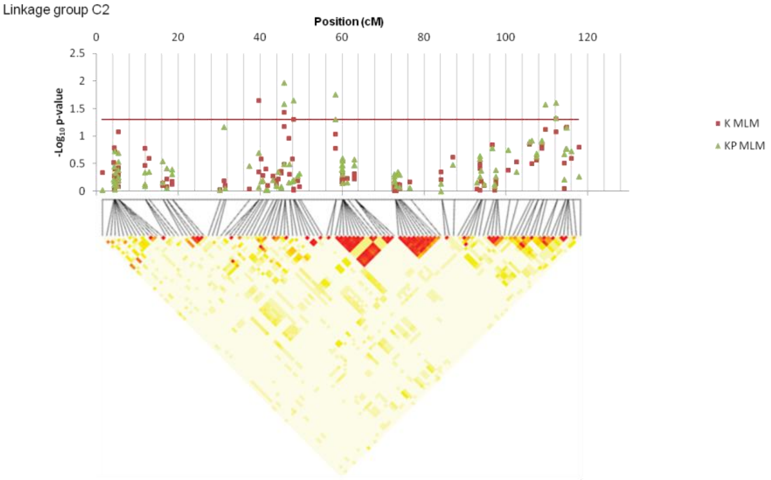
Linkage group C2
Position (cM)
-Log10 p-value
K MLM
KP MLM

Linkage group C3

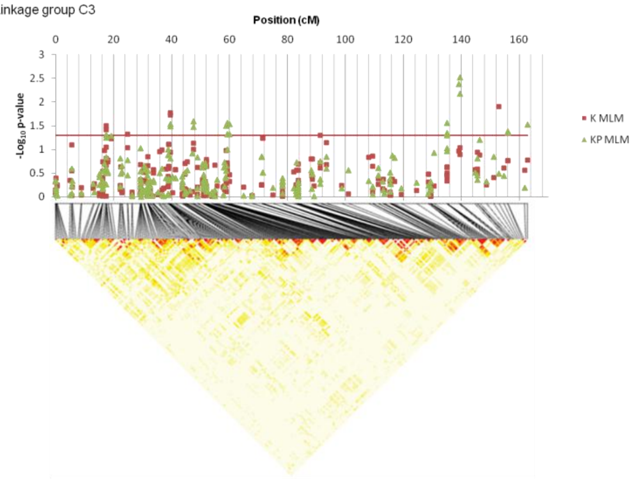

Linkage group C4

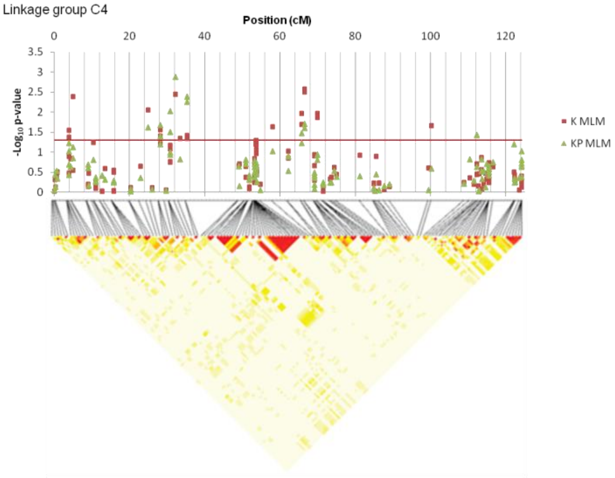

Linkage groupe C5

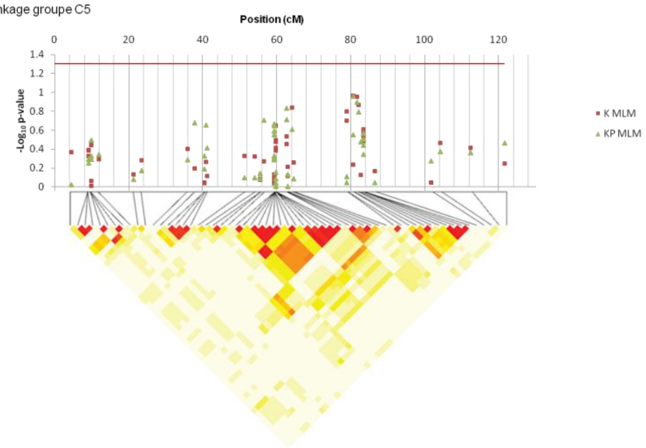

Linkage group C6

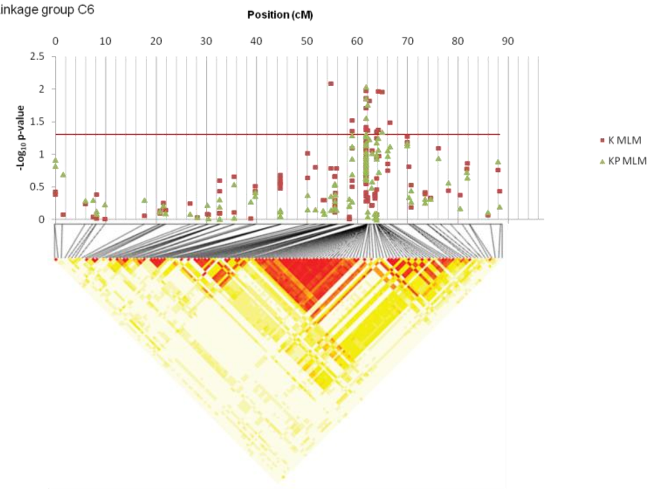

Linkage group C7

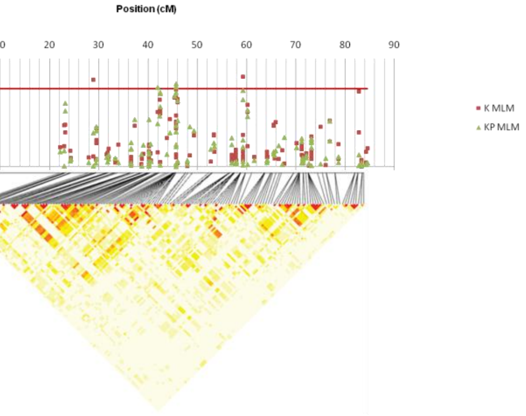

Linkage group C8

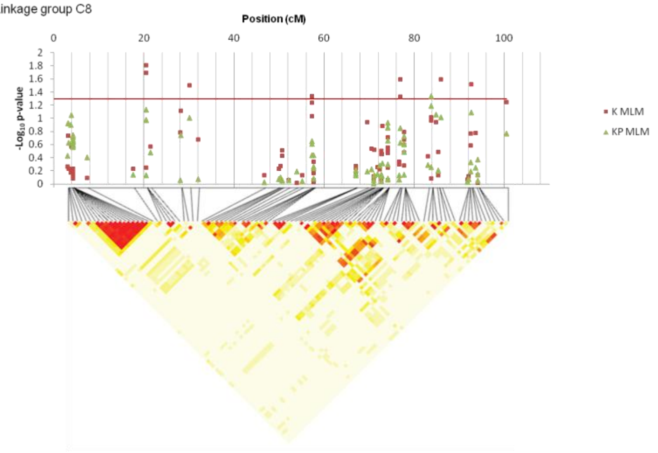

Linkage group C9

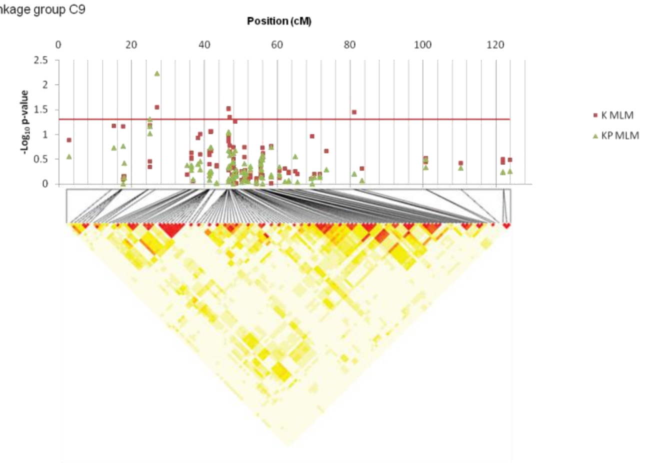

Supplement: Supplementary file 3 — Additional file 3: Figure S2: The linkage disequilibrium pattern and results for association analysis of resistance to stem canker for each linkage group. Negative log10 p values obtained from the K and KP CML models were plotted against the genetic distance (in cM) for each linkage group. The corresponding linkage disequilibrium pattern calculated between pairs of tested markers for association is presented below the Manhattan plot. The more the colour is closer to red, the higher the linkage disequilibrium is. (PDF 1 MB) [file 12864_2013_6190_MOESM3_ESM.pdf]
